# *Porphyromonas gingivalis *and the pathogenesis of rheumatoid arthritis: analysis of various compartments including the synovial tissue

**DOI:** 10.1186/ar4243

**Published:** 2013-06-18

**Authors:** Michele Ciro Totaro, Paola Cattani, Francesco Ria, Barbara Tolusso, Elisa Gremese, Anna Laura Fedele, Sara D'Onghia, Simona Marchetti, Gabriele Di Sante, Silvia Canestri, Gianfranco Ferraccioli

**Affiliations:** 1 Division of Rheumatology, Institute of Rheumatology and Affine Sciences, Catholic University of the Sacred Heart, Via G. Moscati 31, Rome, 00168, Italy; 2 Laboratory of Clinical analyses, Association Columbus, Catholic University of the Sacred Heart, Via G. Moscati 31, Rome, 00168, Italy; 3 Institute of General Pathology, Catholic University of the Sacred Heart, Largo F. Vito 1, Rome, 00168, Italy

## Abstract

**Introduction:**

We evaluated the presence of *Porphyromonas gingivalis *(Pg) DNA in the synovial tissue through synovial biopsy and in other compartments of rheumatoid arthritis (RA) patients in comparison with patients affected by other arthritides. Possible links with clinical, immunologic and genetic features were assessed.

**Methods:**

Peripheral blood (PB), sub-gingival dental plaque, synovial fluid (SF) and synovial tissue samples were collected from 69 patients with active knee arthritis (32 with RA and 37 with other arthritides, of which 14 had undifferentiated peripheral inflammatory arthritis - UPIA). Demographic, clinical, laboratory and immunological data were recorded. The presence of Pg DNA was evaluated through PCR. The HLA-DR haplotype was assessed for 45 patients with RA and UPIA.

**Results:**

No differences arose in the positivity for Pg DNA in the sub-gingival plaque, PB and SF samples between RA and the cohort of other arthritides. Full PB samples showed a higher positivity for Pg DNA than plasma samples (11.8% vs. 1.5%, *P *= 0.04). Patients with RA showed a higher positivity for Pg DNA in the synovial tissue compared to controls (33.3% vs. 5.9%, *P *<0.01). UPIA and RA patients carrying the HLA DRB1*04 allele showed a higher positivity for Pg DNA in the synovial tissue compared to patients negative for the allele (57.1% vs. 16.7%, *P *= 0.04). RA patients positive for Pg DNA in the sub-gingival plaque had a lower disease duration and a higher peripheral blood leucocyte and neutrophil count. The presence of Pg DNA did not influence disease activity, disease disability or positivity for autoantibodies.

**Conclusions:**

The presence of Pg DNA in the synovial tissue of RA patients suggests a pathogenic role of the bacterium. The higher positivity of Pg DNA in full peripheral blood and synovial tissue samples compared to plasma and synovial fluid suggests a possible intracellular localization of Pg, in particular in patients positive for HLA-DR4.

## Introduction

Rheumatoid arthritis (RA) is a chronic systemic inflammatory autoimmune disease. Reasons for the loss of immunologic tolerance against auto-antigens are partially unknown. Genetic factors are thought to be responsible only for 50 to 60% of the RA heritability [1]. HLA alleles DRB1*01 and *04, the main genetic risk factors for RA, are primarily involved in the antigen presentation process [2]. Environmental factors, such as smoking, together with genetic factors, play roles in the development of RA, possibly through the loss of tolerance to citrullinated peptides [3]. Citrullination is a process involved in the pathogenesis of RA, and the conversion of arginine to citrulline allows for a high affinity antigen interaction with the HLA-DRB1*04 allele [4]. Bacteria may play a role as well, activating the immune system against auto-antigens, through the mechanism of the so-called "molecular mimicry" [5]. Following the model of reactive arthritis [6], the research into bacterial antigens in the joint compartment of RA patients resulted in a few positive results, mainly in the synovial tissue compared to the synovial fluid [7], thus supporting the idea of a prevalence of intracellular bacteria. Live bacteria have never been isolated, to date, in the joint compartment of RA patients. The role of these bacterial structures is unknown; they can be bystanders related to the recruitment of inflammatory cells, or they may play a specific part in the etiology of the disease.

Recently, *Porphyromonas gingivalis *(Pg), a periodontal anaerobic intracellular pathogen [8], has been associated with RA and the pathogenesis of the disease [9,10], mainly for the ability of the bacterium to citrullinate host and bacterial peptides [11], unique in the prokaryotic world. Periodontitis is known to be epidemiologically associated with RA [12], and its pathogenesis, including the genetic background, has many points in common with RA [13]. As a support for the possible role of Pg in the pathogenesis of RA, antibodies against Pg have been found to be associated with RA and with anti-citrullinated protein antibodies (ACPA) [14]. Moreover, the DNA of Pg has been detected in the synovial fluid and plasma samples from patients with RA [15], and the coexistence of RA and periodontitis increased the probability of finding Pg DNA in these compartments [16]. Several studies on animal models seem to strengthen the idea of a relationship between Pg and the pathogenesis of RA [17-20], although possible biases should be taken into account.

To date, given the mainly intracellular life of Pg, there are no data regarding the presence of the bacterium in the synovial tissue of RA patients, nor concerning the possible link with clinical and genetic factors. The aims of the study were to see whether Pg DNA could be found in the synovial tissue of RA patients and compare it to patients affected by other arthritides, to determine whether the prevalence of DNA positivity in other compartments, such as dental plaque and peripheral blood of RA patients, could differ from that of patients with other arthritides, and to assess the possible links between the presence of Pg DNA and clinical, immunological and genetic features.

## Methods

### Patients: case-control study

Sixty-nine consecutive patients with active knee arthritis undergoing synovial biopsy from October 2010 until February 2012 at the Division of Rheumatology of the Catholic University of the Sacred Heart of Rome have been included in this study. Twenty-six healthy donors were included in the study, as well. The ethical approval for the study was obtained from the Catholic University of the Sacred Heart Ethical Committee. Informed written consent was obtained from all the patients and healthy donors. The research is in compliance with the Helsinki Declaration. The following samples were collected for every patient: a) peripheral blood, b) sub-gingival dental plaque as previously described [16], c) synovial fluid through joint aspiration, and d) synovial tissue through ultrasound-guided percutaneous needle synovial biopsy (14G Tru-cut Precisa 1410 - Hospital Service). Thirty-two (46.4%) RA patients fulfilling at least six of the American College of Rheumatology/European League Against Rheumatism (ACR/EULAR) criteria for RA [21] were considered as the case population. The control group consisted of 37 subjects with other arthritides. Moreover, samples of peripheral blood and sub-gingival dental plaque were collected from healthy donors.

### Laboratory and clinical data

All patients' sera were tested for the presence of ACPA (Axis-Shield Diagnostics, Dundee, UK), IgM and IgA-rheumatoid factor (RF) autoantibodies (Orgentec diagnostika, Mainz, Germany for IgM and IgA RFs). IgM and IgA RF were considered positive if values were ≥20 U/ml; ACPA were positive if ≥5 U/ml. Patients were tested for erythrocyte sedimentation rate (ESR), C-reactive protein (CRP), complete blood count and synovial fluid cell count, as well. Tender and swollen joint count and the Ritchie articular index were obtained, as well as pain visual analogue scale, global health status and health assessment questionnaire disability index (HAQ-DI). The disease activity score (DAS) was calculated in order to assess the disease activity of patients with rheumatoid arthritis. The presence of joint erosions was evaluated through conventional hand and foot radiograms. Other clinical variables (for example, smoking) were obtained through medical history investigation. Actual therapies with conventional and/or biologic disease modifying anti-rheumatic drugs (DMARDs) and/or steroid were recorded.

### Human leukocyte antigen-DR (HLA-DR) assessment

DNA was extracted by a blood sample through QuickGene DNA Whole Blood kit (Life Science, Fujifilm Corporation, Tokyo, Japan). The extracted DNA was used for molecular typing of the HLA-DR haplotype at allele group level, using the Inno-LiPA HLA-DRB1 Amp Plus kit (Innogenetics N.V., 9052 Gent, Belgium), according to the manufacturer's instructions.

### Histological analysis of the synovial tissue

Histological examination of the synovial tissue samples through direct optical microscopy was performed on 43 of the studied patients (62.3%). The other samples were excluded due to the lack of informativeness. Tissue specimens were fixed with formaldehyde, dehydrated with alcohol, diaphanized with xylol, included in paraffin, sectioned in slices of approximately 10 µm, applied on a slide and then colored with hematoxylin-eosin. A Leica DM 2000 optical microscope has been used for the analysis (Leica Microsystems GmbH, Wetzlar, Germany). Bioptic samples were evaluated by two independent assessors. A binary score was used in order to classify the infiltrate patterns as aggregate or diffuse [22].

### *Porphyromonas gingivalis *DNA detection

Nucleic acid isolation was performed after resuspension of sub-gingival dental plaque samples and shredding of the synovial tissue specimens; all the samples were incubated for 30 minutes at 37°C with a lysis solution and lysozyme (5 mg/ml), and then for 15 minutes at 56°C with the addition of proteinase K. DNA was extracted using a QIAmpDNA Minikit (Qiagen SpA, Milan, Italy) according to the manufacturer's instructions. The presence of Pg DNA was tested through polymerase chain reaction (PCR). Briefly, oligonucleotides 5'-AGG CAG CTT GCC ATA CTG CG-3' and 5'-ACT GTT AGC AAC TAC CGA TGT-3' were used as primers for a 404 bp amplicon derived from the 16S rRNA, as previously described [23]. Each assay used 5 µl of eluted nucleic acids and 20 pmol of each primer. The reaction mixture contained 25 µl of HotStart Taq Master Mix (Qiagen S.p.A., Milan, Italy) and 10 μl of RNase-free water in a final volume of 50 μl. The amplification profile consisted of 15 minutes at 95°C to activate the HotStart-Taq DNA polymerase (Qiagen S.p.A.) followed by 40 cycles at 95°C for 30 sec, 60°C for 45 sec and 72°C for 45 sec. Assays were performed with an iCycler Thermal cycler (Bio-Rad Laboratories, Inc., Hercules, CA, USA). Amplicons were detected by electrophoresis in a 2% agarose gel, visualized by ethidium bromide staining. In each amplification run, distilled water was used as a negative control and genomic DNA from ATCC 33277 strain as a positive control. Procedures to prevent specimen contamination and PCR carryover were rigorously observed at all stages. To assess sensitivity, specific PCR products were cloned into the pCR2.1 vector (Invitrogen, San Diego, CA, USA) and quantified by optical density (OD) measurement. Constructs were serially diluted and subsequently amplified with Pg-specific primers. PCR for *P. gingivalis *was shown to detect concentrations as low as five copies per sample. Randomly selected samples were retested for the presence of *P. gingivalis *DNA, using a real-time PCR Standard kit (Genesig, Eppendorf, England, UK) according to the manufacturer's directions.

### Statistical analysis

Data were analyzed using IBM SPSS Statistics 20.0 (IBM Corp., Armonk, NY, USA) and Prism software 5.0 (Graph-Pad, San Diego, CA 92121-USA).

Categorical and quantitative variables were respectively described as numbers, percentages (%) and mean ± standard deviation (SD). Mann-Whitney's test was used to compare continuous variable. Categorical variables were analyzed using χ^2 ^test or Fisher's test, depending on sample size restrictions.

The concordance among the PCR and real-time PCR tests results was evaluated by Cohen's kappa statistic, as described by Fleiss [24].

A value of *P *<0.05 was considered statistically significant.

## Results

### Patients' clinical and immunological characteristics

Demographic and clinical data of the 32 patients with RA, 14 with undifferentiated peripheral inflammatory arthritis (UPIA) and 23 with other arthritides included in this study, are summarized in Table 1. Four RA patients withdrew the consent for DNA analysis after having obtained the informed consent.

**Table 1:** Patients' clinical and immunological characteristics

	*RA*	*UPIA*	*Others*	*p^a^*	*p^b^*	*p^c^*
*Number*	32	14	23	-	-	-
*Age, mean ± SD (years)*	54.2 ± 17.9	47.8 ± 13.1	47.8 ± 16.7	ns	ns	ns
*N. females/males, (% F)*	24/8 (75.0)	11/3 (78.6)	14/9 (60.9)	ns	ns	ns
*Duration, mean ± SD (years)*	6.5 ± 7.5	3.6 ± 5.3	3.8 ± 6.2	ns	ns	ns
*ESR, mean ± SD (mm 1^st^h)*	62.1 ± 39.2	33.6 ± 26.8	35.9 ± 30.9	**0.02**	**0.01**	ns
*CRP, mean ± SD (mg/l)*	40.7 ± 47.4	15.6 ± 16.9	28.6 ± 49.0	ns	ns	ns
*DAS, mean ± SD*	3.74 ± 1.17	2.25 ± 0.85		**<0.01**	-	-
*HAQ-DI, mean ± SD*	1.40 ± 0.78	0.69 ± 0.91	0.91 ± 0.72	**0.01**	**0.04**	ns
*ACPA ≥5.0 U/ml (%)*	13 (40.6)	1 (7.7)	1 (4.5)	**<0.01**	**<0.01**	ns
*IgM RF ≥20.0 U/ml (%)*	9 (28.1)	2 (15.4)	1 (4.5)	ns	**0.05**	ns
*IgA RF ≥20.0 U/ml (%)*	6 (18.8)	1 (7.7)	0	ns	ns	ns
*DMARDs therapy, n. (%)*	27 (84.4)	8 (57.1)	19 (82.6)	ns	ns	ns
*Biologic therapy, n. (%)*	10 (31.3)	0	2 (8.7)	**0.02**	**0.05**	ns
*Steroid therapy, n. (%)*	22 (68.8)	6 (42.9)	12 (52.2)	ns	ns	ns

CRP, C-reactive protein; DAS, disease activity score; ESR, erythrocyte sedimentation rate; GH, global health; HAQ-DI, health assessment questionnaire disability index; ns, not significant; Others, other arthritides; RA, rheumatoid arthritis; SD, standard deviation; UPIA, undifferentiated peripheral inflammatory arthritis; VAS, visual analogue scale. a = RA vs. UPIA, b = RA vs. Others, c = UPIA vs. Others.

Cases and controls were comparable for age, sex and smoking status. Nine of the RA patients (28.1%) had an early-occurring disease (ERA) with a disease duration of less than one year. The patients with other arthritides comprised 12 patients with seronegative spondyloarthropaties (SpA) and 11 patients with other forms of arthritis (that is, gout, polymyalgia rheumatica, Sjögren syndrome, systemic lupus erythematosus, eosinophilic fasciitis, adult-onset Still's disease, osteoarthritis).

RA patients had a high disease activity and a high disability at the moment of the biopsy (mean DAS 3.74 ± 1.17, mean HAQ-DI 1.40 ± 0.78). Disease activity and disability were higher in RA patients than in patients affected by UPIA (*P *<0.01). A total of 15 (48.4%) RA patients and 3 (25.0%) UPIA patients were positive for at least one tested autoantibody (ACPA, IgM and IgA RF). The presence of joint erosions was significantly higher in RA patients compared to UPIA (63.3% vs. 25.0%, *P *= 0.01) and to patients affected by other arthritides (22.7%, *P *<0.01 vs. RA patients). ERA patients had a lower prevalence of erosions compared to patients with a long-standing disease (LSRA) (22.2% vs. 81.0%, *P *<0.01). Groups were comparable for DMARDs and steroid therapy, although RA patients were more frequently receiving biologic therapy (RA vs. other arthritides: *P *<0.01) (Table 1).

### *Porphyromonas gingivalis *DNA detection

The analysis of the healthy donors showed a positivity for Pg DNA in the sub-gingival dental plaque in seven subjects (26.9%). No positivity arose in the peripheral blood of healthy subjects.

Positive results for Pg DNA in the patients' population are summarized in Table 2 and Additional file 1, Table S1. When considering the global positivity for Pg DNA in all the compartments analyzed, no differences arose among cases and controls.

**Table 2:** Positive results for *Porphyromonas gingivalis *DNA

	*RA*	*UPIA*	*Others*
***Total***	22/31 (71.0)	11/14 (78.6)	16/21 (76.2)
***Plasma***	1/31 (3.2)	0/14	0/21
***Full peripheral blood***	2/21 (9.5)	3/12 (25.0)	1/18 (5.6)
***Synovial fluid***	2/30 (6.7)	1/12 (8.3)	1/20 (5.0)
***Synovial tissue***	9/27 (33.3)	0/11	2/23 (8.7)
***Plaque***	14/26 (53.8)	10/13 (76.9)	11/21 (52.4)

All the values are considered as number of positives/total number (%). Others, other arthritides; RA, rheumatoid arthritis; UPIA, undifferentiated peripheral inflammatory arthritis

When considering the single areas analyzed, 59.3% of all the patients studied were positive for Pg DNA in the sub-gingival plaque, 20.9% were positive in the joint compartment, and only one patient, (affected by LSRA), was positive in the plasma sample. No significant differences arose among patients and healthy donors.

Pg DNA was more present in whole peripheral blood samples than in plasma samples (11.8% vs. 1.5%, *P *= 0.04).

When considering possible differences among cases and controls, no distinction arose in the positivity for Pg DNA in the sub-gingival plaque, plasma and synovial fluid samples (Table 2). Nevertheless, when analyzing the synovial tissue, the positivity for Pg DNA was significantly different among the subjects analyzed (χ^2 ^= 8.05, *P *= 0.02). In particular, patients with RA showed a higher positivity of Pg DNA in the synovial tissue compared to controls (33.3% in RA vs. 5.9% in UPIA + others, *P *<0.01) and also when compared to UPIA (0%, *P *= 0.04 vs. RA patients) or other arthritides (8.7%, *P *<0.01 vs. RA patients), whereas the difference was not significant when comparing RA only to SpA patients (16.7% in SpA, *P *= ns vs. RA patients). No differences arose among LSRA and ERA patients (*P *= ns), though patients with UPIA and ERA showed a trend for a higher positivity for Pg DNA in the sub-gingival plaque than patients with LSRA (75% vs. 47.4%, *P *= 0.1).

These results were replicated using the real-time PCR Standard kit. The concordance between the PCR and the real-time PCR tests results was good (84.8%, Kappa = 0.67, *P *<0.001, 95% CI 0.54 to 0.80).

### Pg DNA positivity and HLA

Forty-five of the 46 patients with RA and/or UPIA were genotyped for HLA DR. DNA extraction and typing of one patient with UPIA were unsuccessful after two attempts. Twenty patients (44.4%) were positive for HLA DRB1*01 and/or DRB1*04 (shared epitope - SE). Thirteen patients (28.9%) were positive for HLA DRB1*01 and 9 (20%) for HLA DRB1*04.

When evaluating a possible association between a specific HLA genotype and the positivity for Pg DNA, no relationship arose among HLA-SE and Pg detection in any of the compartments analyzed. However, when considering the single alleles (DRB1*01 or DRB1*04), UPIA and RA patients carrying HLA DRB1*04 allele showed a higher positivity for Pg DNA in the synovial tissue compared to patients negative for the allele (57.1% vs. 16.7%, *P *= 0.04). The association between the presence of HLA DRB1*04 and the positivity for Pg DNA was confirmed also when considering the whole articular compartment (synovial fluid and synovial tissue) (joint Pg positivity: 55.6% in HLA DRB1*04 positive patients vs. 17.6% in HLA DRB1*04 negative patients, *P *= 0.03). The same trend occurred when considering only patients affected by RA (joint Pg DNA positivity: 55.6% in HLA DRB1*04 positive patients vs. 21.7% in HLA DRB1*04 negative ones) (Additional file 2, Figure S1).

When considering a possible association between clinical variables, HLA and Pg positivity, UPIA and RA patients who were smokers and/or positive for ACPA autoantibodies and/or positive for HLA DRB1*04 allele were more frequently positive for Pg DNA in the synovial tissue, compared to patients who were negative for all these clinical/genetic variables (42.9% vs. 10.5%, *P *= 0.05) (Additional file 3, Table S2).

### Pg DNA positivity and autoantibodies

When evaluating patients affected by RA, no significant relationship arose between the presence of Pg DNA and the positivity for the tested autoantibodies (ACPA, IgM and IgA RF). However, RA patients positive for Pg DNA in the synovial fluid showed a trend for a higher positivity for IgM RF, compared to patients negative for Pg DNA (100% vs. 25.0%, *P *= 0.08). No relationship arose between the positivity for Pg DNA in any of the compartments analyzed and the title of autoantibodies in seropositive RA patients.

### Pg DNA positivity and clinical characteristics

When analyzing the possible links between Pg DNA and the clinical variables in patients affected by RA, we found that patients positive for Pg DNA in the sub-gingival plaque had a shorter disease duration (Pg positive 4.2 ± 5.9 years vs. Pg negative 9.3 ± 8.4 years, *P *= 0.03), and tended to be more commonly smokers compared to patients negative for Pg in the plaque sample (Pg positivity: smokers 88.9% vs. non-smokers 54.5%, *P *= 0.07). No differences arose regarding the other clinical variables analyzed. In particular, no differences were noted in the activity and disability indexes (DAS and HAQ-DI) among RA patients positive or negative for Pg DNA.

**Figure 1:**
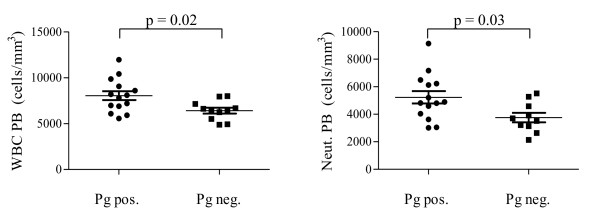
**White blood and blood neutrophils count positivity for *Porphyromonas gingivalis *in the subgingival plaque**. Blood cell count according to Porphyromonas gingivalis positivity in the sub-gingival plaque of RA patients. Pb, peripheral blood; Pg, *Porphyromonas gingivalis; *Neut., neutrophils; WBC, white blood cells.

The presence of Pg DNA in the sub-gingival plaque was associated with modifications in the cell blood count: RA patient positive for Pg DNA had a higher leucocyte and neutrophil count compared to patients negative for the bacterium (leucocytes: Pg positive RA 8.04 ± 1.82 cells × 10^9^/L vs. Pg negative RA 6.41 ± 1.05 cells × 10^9^/L, *P *= 0.02; Neutrophils: Pg positive RA 5.23 ± 1.69 cells × 10^9^/L vs. Pg negative RA 3.76 ± 1.09 cells × 10^9^/L, *P *= 0.03) (Figure 1).

No relationship arose between Pg DNA positivity and synovial fluid cell count.

### Pg DNA and the histological pattern of the synovial tissue

Thirteen patients (30.2%) presented an aggregate organization of the synovial tissue, whereas the other 30 (69.8%) had a diffuse infiltrate. An aggregate pattern was more frequent among RA patients (aggregate in RA: 76.9% vs. aggregate in other patients: 23.1%, *P *= 0.04) and was associated with the positivity for ACPA (ACPA positivity: aggregate 61.5% vs. diffuse 14.3%, *P *= 0.004), for IgM RF (IgM RF positivity: aggregate 46.2% vs. diffuse 10.7%, *P *= 0.02) and for IgA RF (IgA RF positivity: aggregate 30.8% vs. diffuse 3.6%, *P *= 0.02). Patients with an aggregate pattern presented a higher neutrophil count in the synovial fluid (SF) than patients with a diffuse infiltrate (SF neutrophils: aggregate 19.75 ± 15.18 cells × 10^9^/l vs. 8.06 ± 9.72 cells × 10^9^/l, *P *= 0.01). An aggregate pattern of the synovial tissue was more frequent among people carriers of HLA shared epitope compared to patients negative for the alleles (50.0% vs. 9.1%, *P *= 0.04).

When evaluating a possible link between a specific structural pattern of the synovial tissue and the positivity for *Porphyromonas gingivalis *DNA, no relationship arose in any of the compartments analyzed.

## Discussion

In the scientific community it is well accepted that *Porphyromonas gingivalis *(Pg) latent-chronic infection might be one determinant of the citrullination process, because of the peptidyl-arginine-deiminase activity that could potentially lead to the loss of tolerance to citrullinated peptides, thus leading to the occurrence of ACPA [11,13]. Previously, it was shown that antibodies against Pg were more frequent in RA (and in periodontitis patients) than in controls [25] and that these antibodies correlated with ACPA. Since a correlation with CRP was also observed, the main conclusion was that this organism could play a role in disease risk and progression of RA. The occurrence of periodontal disease in many patients with RA is also well established and there is evidence that Pg bacteria induce inflammatory cytokines and proteases by either mononuclear cells or fibroblasts [26,27]. Therefore, Pg can also contribute to the systemic inflammation.

Here we show that Pg DNA can be found in the sub-gingival plaque and in saliva, and rarely, yet possibly, in the peripheral blood mononuclear cells (PBMCs) and even more rarely in plasma, but more frequently in the joint compartment (in the synovial tissue more than in the synovial fluid). Our data support the possibility that the genetic material was carried from teeth to joints [28].

The possibility that oral bacteria or their genetic material could reach the joints was proven by Moen *et al. *[15]. These authors found more genetic material in the synovial fluid than in plasma, but they observed no relationship with the parameters of the inflammatory status (no correlation with CRP, white blood cells count, platelets). Therefore, the possibility of a migration from the oral to the joint compartment was hypothesized, though bacteria seemed to have no link with inflammation. Martinez-Martinez *et al. *[16] confirmed the possibility of a transport of oral genetic material to the joints, since they found Pg among various oral pathogens more frequently in the synovial fluid than in serum and discussed the possibility of a free DNA transport to the joint compartment.

Our data shed more light on the issue, since we observed that Pg DNA is present similarly in UPIA and RA, it is found in the whole blood (of interest more in UPIA than in RA), more rarely in plasma and in the synovial fluid, but it can be found in the synovial bioptic tissue in one-third of RA patients. Although the sample size lacks the power for further assumptions, we also observed that the presence of Pg DNA associates with an increased PBMCs count in the peripheral blood, mostly with polymorphonuclear (PMN) cells, thus possibly contributing to the inflammatory status.

We agree with previous papers that no strict relationship exists between the presence of Pg DNA and ACPA or RF levels [29]. Notably, the Pg DNA presence in the oral compartment was mostly observed in early RA patients rather than in long standing ones, in accordance with the recent findings by J.U. Scher *et al. *[29]. Furthermore, oral Pg DNA was associated with the DR4 epitope. This suggests that the persistence in ERA possibly relies on a defective clearance of Pg by RA patient DR4 carriers. This, of course, should be tested and verified *in vitro *and in further cohorts. The well-defined immunostimulatory properties of Pg DNA, known to stimulate macrophages and fibroblasts to produce tumor necrosis factor alpha and interleukin-6 in a dose-dependent manner, might explain why its persistence could induce a more inflammatory intra-articular inflammation. These stimulatory effects are due to unmethylated CpG motifs within the bacterial DNA and may represent a continuous and persistent inflammatory loop [30].

## Conclusions

Albeit our data cannot support that the presence of *Porphyromonas gingivalis *DNA represents a surrogate marker for the occurrence of ACPA [31], certainly the presence of Pg DNA in UPIA and its presence in the synovial tissue of RA patients, suggests that the bacterium has a role in the pathogenesis of RA, which can still be maintained long after the initial disruption of the immunologic tolerance.

## Abbreviations

ACPA: anti-citrullinated protein antibodies; CRP: C-reactive protein; DAS: Disease Activity Score; DMARDs: disease modifying anti-rheumatic drugs; ERA: early rheumatoid arthritis; ESR: erythrocyte sedimentation rate; HAQ-DI: Health Assessment Questionnaire - Disability Index; HLA: human leukocyte antigen; LSRA: long-standing rheumatoid arthritis; PAD: peptidyl-arginine-deiminase; PBMCs: peripheral blood mononuclear cells; PCR: polymerase chain reaction; Pg: *Porphyromonas gingivalis; *PMN: polymorphonuclear; RA: rheumatoid arthritis; RF: rheumatoid factor; SD: standard deviation; SpA: seronegative spondyloarthropaties; UPIA: undifferentiated peripheral inflammatory arthritis

## Competing interests

The authors declare that they have no competing interests.

## Authors' contributions

MCT performed the experiments, collected patients' data, analyzed the data and wrote the manuscript. PC conceived and designed the experiments, performed the experiments, analyzed the data, contributed reagents/materials/analysis tools, wrote the manuscript. FR performed the experiments and contributed reagents/materials/analysis tools. BT performed the experiments and analyzed the data. EG and ALF collected the patients' data. SD, SM, GDS and SC performed the experiments. GF conceived and designed the experiments, performed the experiments, analyzed the data, contributed reagents/materials/analysis tools and wrote the manuscript. All authors have read and approved the final manuscript for publication.

## Supplementary Material

Additional file 1 **Table S1**. Patients list with demographic, genetic and immunologic data and positivity for *Porphyromonas gingivalis*. Click here for file

Additional file 2 **Figure S1**. Positivity for *Porphyromonas gingivalis *DNA in the synovial tissue and HLA alleles. Click here for file

Additional file 3 **Table S2**. Positivity for *Porphyromonas gingivalis *DNA in the synovial tissue, ACPA positivity, smoking status and HLA DRB1*04 allele. Click here for file

## References

[B1] MacGregor AJ Snieder H Rigby AS Koskenvuo M Kaprio J Aho K Silman AJ Characterizing the quantitative genetic contribution to rheumatoid arthritis using data from twins Arthritis Rheum 2000 15 30 37 10.1002/1529-0131(200001)43:1<30::AID-ANR5>3.0.CO;2-B 10643697

[B2] Hill JA Southwood S Sette A Jevnikar AM Bell DA Cairns E Cutting edge: the conversion of arginine to citrulline allows for a high-affinity peptide interaction with the rheumatoid arthritis-associated HLA-DRB1 *0401 MHC class II molecule J Immunol 2003 15 538 541 12847215 10.4049/jimmunol.171.2.538

[B3] Padyukov L Silva C Stolt P Alfredsson L Klareskog L A gene-environment interaction between smoking and shared epitope genes in HLA-DR provides a high risk of seropositive rheumatoid arthritis Arthritis Rheum 2004 15 3085 3092 10.1002/art.20553 15476204

[B4] Hill JA Southwood S Sette A Jenikar AM Bell DA Cairns E The conversion of arginine to citrulline allows for a high-affinity peptide interaction with the rheumatoid arthritis associated HLA-DRB1*0401 MHC class II molecule J Immunol 2003 15 538 541 12847215 10.4049/jimmunol.171.2.538

[B5] Oldstone MB Molecular mimicry and autoimmune disease Cell 1987 15 819 820 10.1016/0092-8674(87)90507-1 3621346

[B6] Wilbrink B van der Heijden IM Schouls LM van Embden JD Hazes JM Breedveld FC Tak PP Detection of bacterial DNA in joint samples from patients with undifferentiated arthritis and reactive arthritis, using polymerase chain reaction with universal 16S ribosomal RNA primers Arthritis Rheum 1998 15 535 543 10.1002/1529-0131(199803)41:3<535::AID-ART20>3.0.CO;2-4 9506582

[B7] van der Heijden IM Wilbrink B Tchetverikov I Schrijver IA Schouls LM Hazenberg MP Breedveld FC Tak PP Presence of bacterial DNA and bacterial peptidoglycans in joints of patients with rheumatoid arthritis and other arthritides Arthritis Rheum 2000 15 593 598 10.1002/1529-0131(200003)43:3<593::AID-ANR16>3.0.CO;2-1 10728753

[B8] Lamont RJ Jenkinson HF Life below the gum line: pathogenic mechanisms of *Porphyromonas gingivalis* Microbiol Mol Biol Rev 1998 15 1244 1263 9841671 10.1128/mmbr.62.4.1244-1263.1998 PMC98945

[B9] Rosenstein ED Greenwald RA Kushner LJ Weissmann G Hypothesis: the humoral immune response to oral bacteria provides a stimulus for the development of rheumatoid arthritis Inflammation 2004 15 311 318 10.1007/s10753-004-6641-z 16245073

[B10] de Smit MJ Brouwer E Vissink A van Winkelhoff AJ Rheumatoid arthritis and periodontitis; a possible link via citrullination Anaerobe 2011 15 196 200 10.1016/j.anaerobe.2011.03.019 21515392

[B11] Wegner N Wait R Sroka A Eick S Nguyen KA Lundberg K Kinloch A Culshaw S Potempa J Venables PJ Peptidylarginine deiminase from *Porphyromonas gingivalis *citrullinates human fibrinogen and α-enolase: implications for autoimmunity in rheumatoid arthritis Arthritis Rheum 2010 15 2662 2672 10.1002/art.27552 20506214 PMC2941529

[B12] Helminen-Pakkala E Laine V The relationship between periodontal findings and articular involvement in a group of subjects suffering from rheumatoid arthritis Proc Finn Dent Soc 1973 15 52 55 4760752

[B13] Mercado FB Marshall RI Bartold PM Inter-relationships between rheumatoid arthritis and periodontal disease J Clin Periodontol 2003 15 761 772 10.1034/j.1600-051X.2003.00371.x 12956651

[B14] Hitchon CA Chandad F Ferucci ED Willemze A Ioan-Facsinay A van der Woude D Markland J Robinson D Elias B Newkirk M Toes RM Huizinga TW El-Gabalawy HS Antibodies to *Porphyromonas gingivalis *are associated with anticitrullinated protein antibodies in patients with rheumatoid arthritis and their relatives J Rheumatol 2010 15 1105 1112 10.3899/jrheum.091323 20436074

[B15] Moen K Brun JG Valen M Skartveit L Eribe EK Olsen I Jonsson R Synovial inflammation in active rheumatoid arthritis and psoriatic arthritis facilitates trapping of a variety of oral bacterial DNAs Clin Exp Rheumatol 2006 15 656 663 17207381

[B16] Martinez-Martinez RE Abud-Mendoza C Patiño-Marin N Rizo-Rodríguez JC Little JW Loyola-Rodríguez JP Detection of periodontal bacterial DNA in serum and synovial fluid in refractory rheumatoid arthritis patients J Clin Periodontol 2009 15 1004 1010 10.1111/j.1600-051X.2009.01496.x 19929953

[B17] Cantley MD Haynes DR Marino V Bartold PM Pre-existing periodontitis exacerbates experimental arthritis in a mouse model J Clin Periodontol 2011 15 532 541 10.1111/j.1600-051X.2011.01714.x 21434962

[B18] Bartold PM Marino V Cantley M Haynes DR Effect of *Porphyromonas gingivalis*-induced inflammation on the development of rheumatoid arthritis J Clin Periodontol 2010 15 405 411 10.1111/j.1600-051X.2010.01552.x 20507365

[B19] Trombone AP Claudino M Colavite P de Assis GF Avila-Campos MJ Silva JS Campanelli AP Ibañez OM De Franco M Garlet GP Periodontitis and arthritis interaction in mice involves a shared hyper-inflammatory genotype and functional immunological interferences Genes Immun 2010 15 479 489 10.1038/gene.2010.13 20428191

[B20] Kinloch AJ Alzabin S Brintnell W Wilson E Barra L Wegner N Bell DA Cairns E Venables PJ Immunization with *Porphyromonas gingivalis *enolase induces autoimmunity to mammalian α-enolase and arthritis in DR4-IE-transgenic mice Arthritis Rheum 2011 15 3818 3823 10.1002/art.30639 21953289

[B21] Aletaha D Neogi T Silman AJ Funovits J Felson DT Bingham CO Birnbaum NS Burmester GR Bykerk VP Cohen MD Combe B Costenbader KH Dougados M Emery P Ferraccioli G Hazes JM Hobbs K Huizinga TW Kavanaugh A Kay J Kvien TK Laing T Mease P Ménard HA Moreland LW Naden RL Pincus T Smolen JS Stanislawska-Biernat E Symmons D Rheumatoid arthritis classification criteria: an American College of Rheumatology/European League Against Rheumatism collaborative initiative Arthritis Rheum 2010 15 2569 2581 10.1002/art.27584 20872595

[B22] Yanni G Whelan A Feighery C Bresnihan B Analysis of cell populations in rheumatoid arthritis synovial tissues Semin Arthritis Rheum 1992 15 393 399 10.1016/0049-0172(92)90040-K 1626285

[B23] Slots J Ashimoto A Flynn MJ Li G Chen C Detection of putative periodontal pathogens in subgingival specimens by 16S ribosomal DNA amplification with the polymerase chain reaction Clin Infect Dis 1995 15 Suppl 2 304 307 10.1093/clinids/20.supplement_2.s304 7548580

[B24] Fleiss JL Statistical Methods for Rates and Proportions 1981 New York: John Wiley & Sons Inc

[B25] Mikuls TR Payne JB Reinhardt RA Thiele GM Maziarz E Cannella AC Holers VM Kuhn KA O'Dell JR Antibody responses to *Porphyromonas gingivalis *(*P. gingivalis*) in subjects with rheumatoid arthritis and periodontitis Int Immunopharmacol 2009 15 38 42 10.1016/j.intimp.2008.09.008 18848647 PMC2748386

[B26] Fagundes JA Monoo LD Euzébio Alves VT Pannuti CM Cortelli SC Cortelli JR Holzhausen M *Porphyromonas gingivalis *is associated with protease-activated receptor-2 upregulation in chronic periodontitis J Periodontol 2011 15 1596 1601 10.1902/jop.2011.110073 21513479

[B27] Belibasakis GN Bostanci N Reddi D Regulation of protease-activated receptor-2 expression in gingival fibroblasts and Jurkat T cells by *Porphyromonas gingivalis* Cell Biol Int 2010 15 287 292 10.1042/CBI20090290 19947912

[B28] Loyola-Rodriguez JP Martinez-Martinez RE Abud-Mendoza C Patiño-Marin N Seymour GJ Rheumatoid arthritis and the role of oral bacteria J Oral Microbiol 2010 15 2 10.3402/jom.v2i0.5784 PMC3084578 21523217

[B29] Scher JU Ubeda C Equinda M Khanin R Buischi Y Viale A Lipuma L Attur M Pillinger MH Weissmann G Littman DR Pamer EG Bretz WA Abramson SB Periodontal disease and the oral microbiota in new-onset rheumatoid arthritis Arthritis Rheum 2012 15 3083 3094 10.1002/art.34539 22576262 PMC3428472

[B30] Nonnenmacher C Dalpke A Zimmermann S Flores-De-Jacoby L Mutters R Heeg K DNA from periodontopathogenic bacteria is immunostimulatory for mouse and human immune cells Infect Immun 2003 15 850 856 10.1128/IAI.71.2.850-856.2003 12540566 PMC145359

[B31] Hitchon CA El-Gabalawy HS Infection and rheumatoid arthritis: still an open question Curr Opin Rheumatol 2011 15 352 357 10.1097/BOR.0b013e3283477b7b 21532483

