# Supplementary material for: Porphyromonas gingivalis and the pathogenesis of rheumatoid arthritis: analysis of various compartments including the synovial tissue
Source: Arthritis Res Ther. 2013 Jun 18;15(3):R66. doi: 10.1186/ar4243 (PMC4060366; doi:10.1186/ar4243)
Supplement: Additional file 2 — Figure S1. Positivity for Porphyromonas gingivalis DNA in the synovial tissue and HLA alleles. [file ar4243-S2.DOC]

**Supplementary Figure 1. Positivity for *Porphyromonas gingivalis* DNA in the synovial tissue and HLA alleles.**


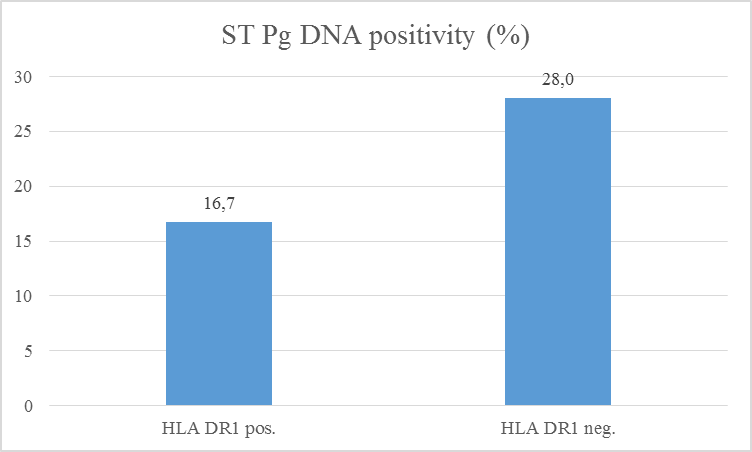


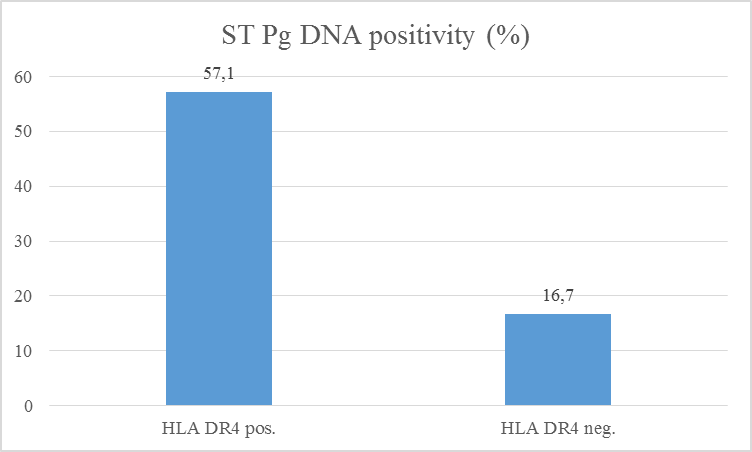


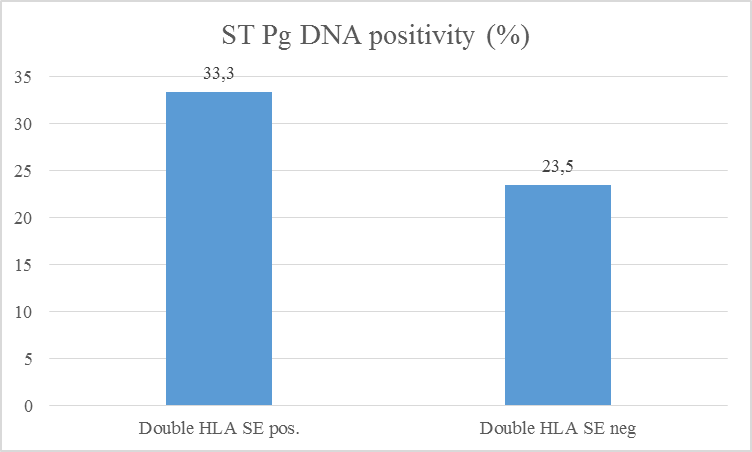


ST: synovial tissue; Pg: *Porphyromonas gingivalis*; Double HLA SE: presence of a double allele of the shared epitope (either DR1 or DR4).
